# What Evidence Underlies Clinical Practice in Paediatric Surgery? A Systematic Review Assessing Choice of Study Design

**DOI:** 10.1371/journal.pone.0150864

**Published:** 2016-03-09

**Authors:** Benjamin Allin, Nicholas Aveyard, Timothy Campion-Smith, Eleanor Floyd, James Kimpton, Kate Swarbrick, Emma Williams, Marian Knight

**Affiliations:** 1 National Perinatal Epidemiology Unit, Oxford, United Kingdom; 2 Department of Paediatric Surgery, Oxford Children’s Hospital, Oxford, United Kingdom; 3 University of Oxford Medical School, Oxford, United Kingdom; 4 Royal Berkshire Hospital NHS Trust, Reading, United Kingdom; 5 Oxford University Hospitals NHS Trust, Oxford, United Kingdom; University Hospital Oldenburg, GERMANY

## Abstract

**Objective:**

Identify every paediatric surgical article published in 1998 and every paediatric surgical article published in 2013, and determine which study designs were used and whether they were appropriate for robustly assessing interventions in surgical conditions.

**Methods:**

A systematic review was conducted according to a pre-specified protocol (CRD42014007629), using EMBASE and Medline. Non-English language studies were excluded. Studies were included if meeting population criteria and either condition or intervention criteria. Population: Children under the age of 18, or adults who underwent intervention for a condition managed by paediatric surgeons when they were under 18 years of age. Condition: One managed by general paediatric surgeons. Intervention: Used for treatment of a condition managed by general paediatric surgeons.

**Main Outcome Measure:**

Studies were classified according to whether the IDEAL collaboration recommended their design for assessing surgical interventions or not. Change in proportions between 1998 and 2013 was calculated.

**Results:**

1581 paediatric surgical articles were published in 1998, and 3453 in 2013. The most commonly used design, accounting for 45% of studies in 1998 and 46.8% in 2013, was the retrospective case series. Only 1.8% of studies were RCTs in 1998, and 1.9% in 2013. Overall, in 1998, 9.8% of studies used a recommended design. In 2013, 11.9% used a recommended design (proportion increase 2.3%, 95% confidence interval 0.5% increase to 4% increase, p = 0.017).

**Conclusions and Relevance:**

A low proportion of published paediatric surgical manuscripts utilise a design that is recommended for assessing surgical interventions. RCTs represent fewer than 1 in 50 studies. In 2013, 88.1% of studies used a less robust design, suggesting the need for a new way of approaching paediatric surgical research.

## Introduction

As highlighted by the Chief Medical Officer’s report *Our Children Deserve Better: Prevention Pays*[1], and the *NHS Atlas of Variation in Healthcare for Children and Young People*[2], significant variation exists across the UK in health outcomes for children. Variation in outcomes and management has been highlighted specifically in many paediatric surgical conditions[3, 4].

Three possible reasons for variation in clinical practice exist; clinician non-adherence to evidence based management guidelines, a lack of translation of high quality evidence into management guidelines, or a failure to develop a robust high-quality evidence base. In 1998, Hardin et al[5] attempted to assess the types of study design that comprised the paediatric surgical evidence base, concluding that it was largely descriptive, and that the need for better outcomes research data in pediatric surgery would require more rigorous study designs and multi-institutional coordination. This study was however limited in its scope, as it only reviewed articles published in the core paediatric surgical literature, therefore potentially excluding more robust studies that were published in higher impact factor journals.

Since Hardin et al conducted their study, there has been a greater emphasis placed on evidence-based practice, both in medicine and in surgery. In 2009, the IDEAL collaboration published a framework for the robust assessment of surgical interventions[6]. This framework, akin to that used for assessment of new pharmaceuticals, is widely promoted as the gold standard tool for use in surgical research. IDEAL suggest that the most appropriate study designs for robustly assessing surgical interventions are prospective cohort studies, randomised controlled trials, and long-term registry studies[6–9]. The overall objective of this review was to identify whether there has been any improvement in the paediatric surgical evidence base since Hardin et al conducted their study. In order to achieve this global objective, we aimed to identify every paediatric surgical manuscript published in 1998 (the year of Hardin’s paper), and every paediatric surgical manuscript published in 2013 (the last year for which complete data is available), and examine and compare what proportion utilised a study design that was recommended by the IDEAL collaboration.

## Methods

The review was conducted according to a pre-specified protocol that was registered on the Prospero International Prospective Register of Systematic Reviews (CRD42014007629). Multiple search strategies were used via the OVID interface to identify relevant articles from Medline and EMBASE. Search terms were identified from database thesauri *(italics)* and free-text, relating to surgery (e.g. Surgery, *Surgical Procedures*), key paediatric surgical conditions (e.g. Oesophageal atresia, *Appendicitis*), key journals (e.g. Annals of Surgery, British Journal of Surgery, Journal of Paediatric Surgery) and Children (e.g. infant, *Infant (very low birth-weight*)), and were combined using Boolean operators. Searches were performed with year limits applied initially to 1998 and then to 2013. Searches were performed on the 7^th^ of March 2014. See appendix 1 for full search strategies.

### Inclusion criteria

Studies were included if they met population criteria, and either condition or intervention criteria. The populations of interest were children under the age of eighteen, or adults who had undergone an intervention (conservative, medical or surgical) for a condition managed by paediatric surgeons, when they were less than eighteen years of age. Conditions of interest were all those managed by general paediatric surgeons, whilst interventions of interest were all those used for treatment of a condition managed by general paediatric surgeons. No conditions were excluded.

For the purposes of this review, ‘general paediatric surgeon’ included all specialists covered by a United Kingdom certificate of completion of training in paediatric surgery. This therefore included paediatric urologists, but excluded paediatric specialists in the following disciplines; plastic surgery, ear nose and throat surgery, orthopaedic surgery, cardiothoracic surgery, maxillo-facial surgery and neurosurgery.

Non-clinical studies, or manuscripts consisting of expert opinion alone were not eligible for inclusion in the review. Due to the large number of papers that were anticipated to be identified through the search strategy, non-English language articles were not considered for inclusion.

### Primary Outcome

The primary outcome was the proportion of paediatric surgical manuscripts published from the 1^st^ of January 1998 to the 31^st^ of December 1998, and from the 1^st^ of January 2013 to the 31^st^ December 2013 that were classified as:

1. Systematic reviews
2. Long-term follow-up studies
3. Randomised controlled trials
4. Prospective cohort studies
5. Case-control studies
6. Retrospective case series/cohort studies
7. Case studies/innovation studies.

Long-term follow-up studies, randomised controlled trials and prospective cohort studies are deemed appropriate for assessment of surgical interventions by the IDEAL collaboration. Case-control, retrospective case series and case studies are not. Systematic reviews fall outside of the recommendations made by the IDEAL collaboration.

Standard definitions of each study design were used. A specific time-period was not used to define ‘long-term’, since this was likely to vary according to the condition being investigated. Where there was disagreement between reviewers as to the definition of type of study, this was resolved by mutual discussion, with recourse to a third reviewer if required.

### Secondary outcome measures

The following secondary outcome measures were considered:

1. The change between 1998 and 2013 in proportion of study designs deemed appropriate by the IDEAL collaboration for assessment of interventions in surgical conditions.
2. The proportion of studies investigating any aspect of each of paediatric appendicitis, pyloric stenosis, Hirschsprung’s disease, gastroschisis, transplantation or oesophageal atresia between the 1^st^ of January 1998 and the 31^st^ of December that use methodologies deemed appropriate by the IDEAL collaboration for assessment of interventions in surgical conditions.
3. The proportion of studies investigating any aspect of each of paediatric appendicitis, pyloric stenosis, Hirschsprung’s disease, gastroschisis, transplantation or oesophageal atresia between the 1^st^ of January 2013 and 31^st^ of December 2013 that use methodologies deemed appropriate by the IDEAL collaboration for assessment of interventions in surgical conditions.

### Study Assessment

Each title was screened for eligibility by one of NA, TCS, EF, JK, KS or EW. BA reviewed a sample of 100 titles excluded by each reviewer to ensure correct application of eligibility criteria. Abstracts, and, if necessary, full papers were then reviewed for each included title by two reviewers working independently to re-confirm eligibility, and assign a study design. Discrepancies were resolved by discussion with a third reviewer (BA).

Smart group automated search strategies within Endnote (Endnote X7.1, Bld 9529, Thomson Reuters, America) were used to identify papers whose title reported that the focus of the paper was paediatric appendicitis, pyloric stenosis, Hirschsprung’s disease, gastroschisis, transplantation or oesophageal atresia. Papers included in these sub-groups were reviewed by BA to ensure that their focus was on the condition of interest.

### Post-hoc analyses

After developing and registering the study protocol, but prior to data extraction, the decision was made to exclude laboratory-based studies. Originally, the intention of the paper was to describe the overall paediatric surgical literature, with the aim of assessing how well it supported clinical decision-making. However, it was felt that laboratory studies contributed little to this overall aim and the decision was made to exclude them.

During data collection, it was noted that a large proportion of eligible studies were focussed on transplantation. Transplantation is only undertaken by a very few paediatric surgeons, and is more often performed by adult surgeons who have sub-specialised in paediatric transplantation. As research into transplantation appeared to be forming a large proportion of the published paediatric surgical literature, the decision was taken to assess the proportion of published manuscripts that focussed on transplantation, as well as conducting a sensitivity analysis to determine whether their exclusion significantly affected outcomes.

### Statistical Analysis

Proportions of utilised study designs were calculated for 1998 and 2013, together with differences in proportions with 95% confidence intervals. Proportions were compared using a χ^2^ test. The proportion of appropriate study designs in 1998 and 2013 was calculated for each of paediatric appendicitis, pyloric stenosis, Hirschsprung’s disease, gastroschisis, transplantation or oesophageal atresia. All statistical analysis was performed using Stata version 13 (StataCorp. 2009. Stata: Release 11. Statistical Software. College Station, TX: StataCorp LP).

## Results

### Characteristics of included studies

**Fig 1:**
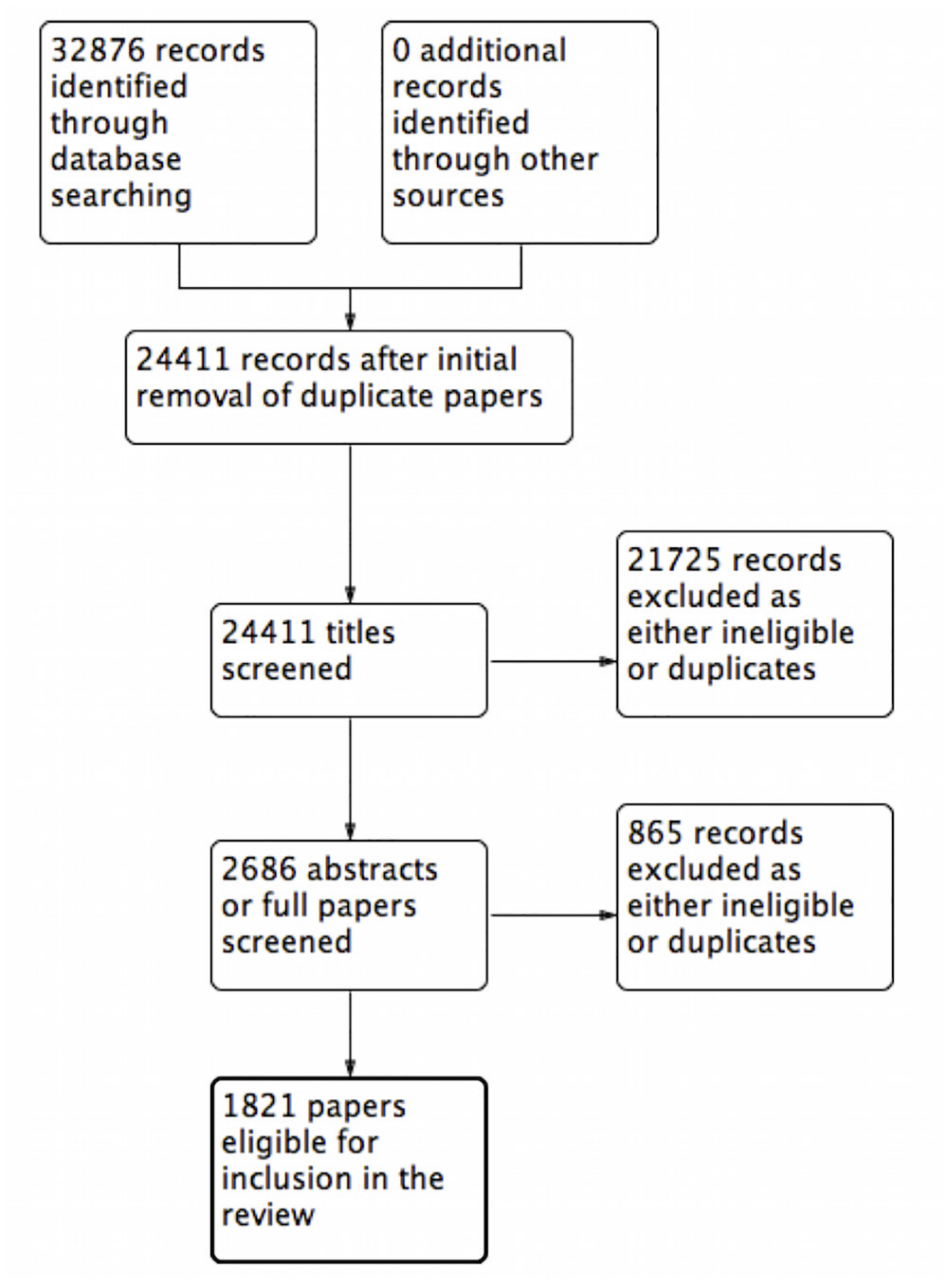
PRISMA flow diagram 1998.

**Fig 2:**
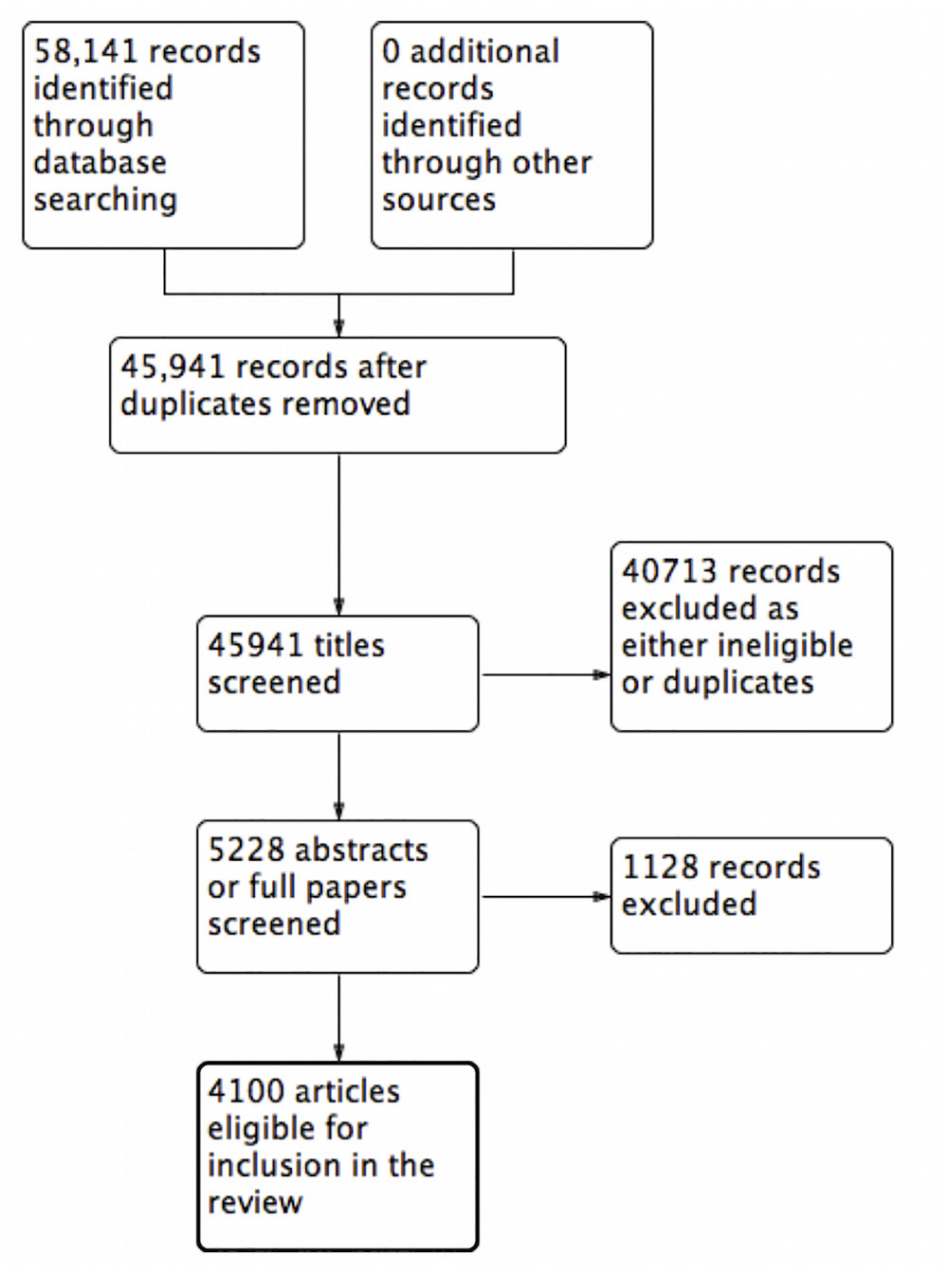
PRISMA flow diagram 2013.

Using the stated search strategy, 32,876 records were identified in 1998. After automated removal of duplicates, 24,411 titles remained for screening. From this, 1821 papers were eventually deemed eligible for inclusion in the review (Fig 1). With the year limit 2013 applied, 58,141 records were identified. After automated removal of duplicates, 45,941 titles remained for screening. From this, 4100 papers were eventually deemed eligible for inclusion in the review (Fig 2).

### Primary outcome measure

With transplant studies included, in both 1998 and 2013, the most commonly used study design was a retrospective case series/cohort study, accounting for 47% and 49.9% of studies respectively. The next most commonly used study design was a single patient case study, accounting for 39% of studies in 1998 and 32.1% in 2013. Randomised controlled trials were only used in 1.8% of studies in 1998 and 1.7% of studies in 2013. When transplant studies were excluded, there was no change in the order of study design from most commonly used to least commonly used, and only very small changes in proportions of each study design were seen (Table 1).

**Table 1:** Proportions of each study design in 1998 and 2013.

	1998		2013	
Types of study	Including transplant studies	Excluding transplant studies	Including transplant studies	Excluding transplant studies
**Less appropriate study designs as per the IDEAL recommendations**				
**Overall**	1620 (89%)	1419 (89.8%)	3497 (85.3%)	2941 (85.2%)
*Case Studies*	710 (39%)	661 (41.8%)	1318 (32.1%)	1222 (35.4%)
*Retrospective case series or retrospective cohort study*	856 (47%)	712 (45%)	2046 (49.9%)	1615 (46.8%)
*Case-Control Study*	54 (3%)	46 (2.9%)	133 (3.2%)	104 (3.0%)
**More appropriate study designs as per the IDEAL recommendations**				
**Overall**	195 (10.7%)	156 (9.8%)#	498 (12.1%)	410(11.9%)#
*Prospective Cohort Studies*	137 (7.5%)	110 (7%)	388 (9.5%)	316 (9.2%)
*Randomised Controlled Trials*	32 (1.8%)	29 (1.8%)	71 (1.7%)	65 (1.9%)
*Long-term follow-up*	26 (1.4%)	17 (1.1%)	39 (1.0%)	29 (0.8%)
**Not classified in IDEAL recommendations**				
**Systematic Review**	6 (0.3%)	6 (0.4%)	105 (2.6%)	102 (2.9%)
**Total**	1821	1581	4100	3453

^#^Difference in proportion utilising appropriate study designs = 2.3% (95% CI 0.5%-4%, p = 0.017). Comparison made excluding transplant studies

### Secondary outcome measures

#### Change in proportions

In 1998, 156 of 1581 studies (9.8%) utilised a design that was deemed appropriate for assessing the efficacy of an intervention in a surgical condition. In 2013, 410 of 3543 studies (11.9%) utilised a design that was appropriate (difference in proportions between 1998 and 2013, 2.3%, 95% confidence interval 0.5%-4.0%, p = 0.017).

#### Studies of individual conditions

Studies of individual conditions reflected the same pattern as the overall results. In 1998, with 0% of identified manuscripts utilising the most appropriate study designs, both gastroschisis and oesophageal atresia research had the lowest proportions of appropriate study design usage. Transplant research had the highest proportion, with 16% of study designs being appropriate. In 2013, published studies of oesophageal atresia had the lowest proportion of appropriate study designs (10%), with Hirschsprung’s disease research having the highest usage of appropriate study designs (18%) (Table 2).

**Table 2:** Proportion of appropriate and inappropriate study designs for key paediatric surgical conditions in 1998 and 2013.

	Number of studies in 1998		Number of studies in 2013
**Appendicitis**			
**Less appropriate designs**	47 (89%)	134 (83%)	
**Appropriate designs**	6 (11%)	23 (14%)	
**Systematic Review**	0 (0%)	4 (2%)	
**Pyloric Stenosis**			
**Less appropriate designs**	22 (92%)	23 (85%)	
**Appropriate designs**	2 (8%)	3 (11%)	
**Systematic Review**	0 (0%)	1 (4%)	
**Hirschsprung’s Disease**			
**Less appropriate designs**	36 (95%)	41 (82%)	
**Appropriate designs**	2 (5%)	8 (16%)	
**Systematic Review**	0 (0%)	1 (2%)	
**Oesophageal atresia**			
**Less appropriate designs**	11 (100%)	35 (90%)	
**Appropriate designs**	0 (0%)	2(5%)	
**Systematic Review**	0 (0%)	2 (5%)	
**Gastroschisis**			
**Less appropriate designs**	12 (100%)	37 (86%)	
**Appropriate designs**	0 (0%)	4 (9%)	
**Systematic Review**	0 (0%)	2 (5%)	
**Transplant**			
**Less appropriate designs**	201 (84%)	556 (86%)	
**Appropriate designs**	39 (16%)	87 (13%)	
**Systematic Review**	0 (0%)	3 (1%)	

### Study Limitations

The review is, by its nature, limited to articles that were published in the two years chosen for investigation, introducing the possibility of any result being influenced by the years that we chose to investigate. However, it is highly unlikely given the number of titles included in the review, and the number of conditions covered that the overall pattern of results seen are due to chance or systematic bias occurring as a result of choosing these two years, 1998 and 2013. There is no reason to suppose that the patterns of studies published would vary substantively between individual years. By undertaking a sub-group analysis of six commonly managed paediatric surgical conditions, and demonstrating that the patterns of study designs utilised are similar to the overall pattern seen, we believe that these results are representative of the current situation of the paediatric surgical evidence base.

## Discussion

This review has shown that, despite more than a doubling in the size of the evidence base between 1998 and 2013, still only a small number of published paediatric surgical studies use optimal designs for assessing interventions in surgical conditions. Randomised controlled trials, which provide the highest level of evidence are utilised in less than two per-cent of studies, and the majority of the evidence-base is built upon retrospective observational studies, which are open to the influence of chance, confounding and bias. Despite the increasing prevalence of the evidence-based medicine movement, this study has also shown that in paediatric surgery there has been little meaningful improvement in study design over the past fifteen years, with only a 2% increase in the proportion of studies using optimal designs.

Recently, Zani-Ruttenstock et al attempted to determine the highest quality of evidence, as determined by the OCEBM levels of evidence[10], that was available to support the treatment of key paediatric surgical conditions[11]. They concluded that 49% of paediatric surgical procedures were supported by level 1a (the highest) evidence. This result has been taken by some to suggest that there is a high quality evidence base available to support clinical practice in paediatric surgery. We do not agree with this. The results of the Zani-Ruttenstock paper are based upon 126 procedures that were undertaken in a one-month period at a large teaching hospital. Fifty-seven of these procedures (45%) were either appendicectomies or inguinal herniotomies. The remaining 55% of procedures performed then encompassed 34 different conditions. The levels of evidence that support treatment of appendicitis and inguinal hernia will therefore unduly influence the results of the study and its conclusions. As both of these conditions are reported to be supported by level 1a evidence, we believe the Zani-Ruttenstock paper significantly over-estimates the overall quality of the evidence-base supporting clinical practice in paediatric surgery. We believe that they have further over-estimated the quality of the evidence base for two key reasons:

1. They have included studies performed in adults in their analysis. There are significant differences in anatomy, physiology, presentation and rehabilitation between children and adults, which mean that we should not automatically generalize the results of studies performed in adults to paediatric populations. When licensing a pharmaceutical product, evidence of its safety and efficacy must have been obtained from paediatric studies. Why should surgical procedures be any different?
2. They have over-estimated the levels of evidence attributed to key studies. This includes those supporting their results for appendicectomy and inguinal herniotomy. The systematic reviews supporting the management of inguinal herniotomy are graded as level 1a evidence (systematic reviews of homogeneous randomized controlled trials). They are in fact systematic reviews of heterogeneous cohort studies, randomised controlled trials, and potentially also case series. They should therefore at best be graded as level 2a-, and potentially 3a-. The OCEBM state that a ‘-‘ suggests that although the study may in theory be a high level of evidence, it fails to provide a conclusive answer. The randomized controlled trial identified as providing evidence for management of appendicitis is a pilot trial, and therefore should not be used to draw conclusions on superiority of treatments, merely to determine the potential for conducting a definitive randomized controlled trial.

For these three key reasons, we believe that undertaking a large scale, robust, systematic review provides a much more accurate assessment of the overall paediatric surgical literature.

Ostlie et al have suggested that as few as 56 paediatric surgical randomized controlled trials were published between 1999 and 2009[12]. Only 19 of these had appropriate randomization, study design and sample sizes. In the key paediatric surgical literature, they report that randomized controlled trials account for only 0.34% of published manuscripts, and that this percentage has little changed since a similar study was conducted in 1999[13]. By making a specific assessment of the quality of the randomized controlled trials that are being undertaken in paediatric surgery, this work further supports our conclusions that existing paediatric surgical research is not sufficient to support clinical practice.

At present, in paediatric surgery, multiple interventions are widely used for relatively common conditions^10-14^. One such example is gastroschisis, in which a recent systematic review identified only 8 studies totalling 804 infants comparing the two most common surgical interventions[14]. Six of these studies were retrospective. The reviews conclusions were that the conducted studies were too heterogeneous in their populations, interventions and outcomes, as well as too likely to have been influenced by bias and confounding to allow any robust conclusions to be drawn. This current review would suggest that this pattern is the norm in paediatric surgery. As Chalmers et al eloquently described in 2014, allowing this situation to persist not only impacts on the quality of care provided to patients, but also results in study participants being exposed to unnecessary risk, delays the implementation of successful interventions, and has significant financial implications for funders and health care providers[15].

It is also not unreasonable to suggest that children are experiencing excess morbidity as a result of the poor quality of the existing evidence base in paediatric surgery. A lack of evidence to show difference in outcomes between two interventions is not the same as evidence of no difference, and it is highly likely therefore that in many paediatric surgical conditions where multiple inadequately evidence-based interventions are used, that one intervention is actually inferior to the other. A sub-set of infants will therefore be treated with an inferior intervention, and will have worse outcomes than they would, had there been sufficient evidence to highlight the difference in outcomes between treatment modalities.

There are multiple reasons for the lack of appropriate study designs being used in paediatric surgery. However, the most important is the low incidence of many of the encountered conditions. This results in individual surgical centres seeing only a handful of children with each condition in a year, making it impossible for surgeons or researchers working within their individual centres to conduct studies with sufficient statistical power to address clinically meaningful questions over a sensible time-period. In order to attempt to increase study population size, many researchers therefore perform retrospective case series over many years, or even many decades, with the requisite introduction of heterogeneity in population and intervention. The alternative method for increasing study population size, collaboration between centres, is currently rarely used in paediatric surgical research.

It is unclear why there is not more multi-centre collaboration in paediatric surgical research. However, if sample populations are to be established that are large enough for the conduct of high quality randomised controlled trials in conditions such as gastroschisis and Hirschsprung’s Disease, it is a status that must be altered. A model similar to that used in neonatal research could be adopted. Neonatal barriers to conduct of randomised controlled trials are similar to those in paediatric surgery; low incidence, ethical considerations regarding consent, and practical considerations surrounding randomisation in emotive settings and blinding to interventions. Many of these issues have been overcome through using a central co-ordinating centre with academic excellence and trials experience to run large-scale, multi-site, national and international randomised controlled trials such as the TOBY[16], TOBY^XE^[17], PIPS[18] and BOOST II UK trials[19, 20]. Expanding studies to involve international collaborators is potentially difficult. However, the model of first establishing a robust national collaboration and then expanding this internationally has been shown to be successful in the INDOSS-Assam project, an Indian expansion of the UK Obstetric Surveillance System upon which BAPS-CASS is based. We would advocate for developing an international paediatric surgical research collaboration that is modelled upon that used by the neonatologists, and expanded in the manner used for INDOSS-Assam[21].

This systematic review has highlighted how the current framework for conducting paediatric surgical research is not allowing the conduct of large numbers of studies using appropriate methodology, and more importantly, is not allowing for improvements to be made in the quality of the paediatric surgical evidence-base. To achieve this, there needs to be a fundamental re-imagining of the way we undertake paediatric academic surgical practice. Such a re-imagining could include the development of a priority setting partnership to identify target areas for research, the use of core outcome sets to ensure patient relevance, and most importantly, enhanced collaboration on a national and international level to ensure that studies are designed with adequate statistical power to demonstrate meaningful differences in outcomes. Only through developing a research network that meets these principles, and integrates existing successful collaborations such as BAPS-CASS and CAPSNet with trainee collaboratives, governing bodies, and routine data collection systems will we be able to conduct the studies the specialty requires. Failure to address the fundamental problems in paediatric surgical research will only result in persistence of the status quo, along with the on-going exposure of patients to potentially inferior treatments and the risk of excess morbidity.

## Supporting Information

S1 PRISMA Checklist (DOC)
